# Molecular phylogenetics of the sucking louse genus *Lemurpediculus* (Insecta: Phthiraptera), ectoparasites of lemurs, with descriptions of three new species

**DOI:** 10.1016/j.ijppaw.2023.02.002

**Published:** 2023-02-09

**Authors:** Andrea Springer, Lance A. Durden, Frederik Kiene, Annette Klein, Romule Rakotondravony, Julian Ehlers, Stephen E. Greiman, Marina B. Blanco, Sarah Zohdy, Sharon E. Kessler, Christina Strube, Ute Radespiel

**Affiliations:** a Institute for Parasitology, Centre for Infection Medicine, University of Veterinary Medicine Hannover, Buenteweg 17, 30559, Hanover, Germany; b Department of Biology, Georgia Southern University, 4324 Old Register Road, Statesboro, GA, 30458, USA; c Institute of Zoology, University of Veterinary Medicine Hannover, Buenteweg 17, 30559, Hannover, Germany; d Clinic for Swine and Small Ruminants, Forensic Medicine and Ambulatory Service, University of Veterinary Medicine Hannover, 30173, Hannover, Germany; e École Doctorale Ecosystèmes Naturels (EDEN), University of Mahajanga, 5 Rue Georges V - Immeuble KAKAL, Mahajanga Be, B.P. 652, Mahajanga 401, Madagascar; f Faculté des Sciences, de Technologies et de l’Environnement, University of Mahajanga, 5 Rue Georges V - Immeuble KAKAL, Mahajanga Be, B.P. 652. Mahajanga 401, Madagascar; g Animal Ecology and Conservation, Institute of Cell and Systems Biology of Animals, University of Hamburg, Martin-Luther-King-Platz 3, 20146, Hamburg, Germany; h Duke Lemur Center, Durham, NC, 27705, USA; i Department of Biology, Duke University, Durham, NC, 27708, USA; j School of Forestry and Wildlife Sciences, 602 Duncan Drive, Auburn, AL, 36849, USA; k Department of Psychology, Faculty of Natural Sciences, University of Stirling, Stirling, FK9 4LA, Scotland, UK

**Keywords:** Anoplura, Cheirogaleidae, Co-speciation, Host specificity, Madagascar, *Microcebus*, COI, cytochrome C oxidase subunit I, EF1α, elongation factor 1α, ITS1, internal transcribed spacer 1, SEM, scanning electron microscopy

## Abstract

Sucking lice live in intimate association with their hosts and often display a high degree of host specificity. The present study investigated sucking lice of the genus *Lemurpediculus* from six mouse lemur (*Microcebus*) and two dwarf lemur (*Cheirogaleus*) species endemic to the island of Madagascar, considered a biodiversity hotspot. Louse phylogenetic trees were created based on cytochrome C oxidase subunit I (COI), elongation factor 1α (EF1α) and internal transcribed spacer 1 (ITS1) sequences. While clustering according to host species was generally observed for COI and ITS1, suggesting high host specificity of the examined lice, EF1α sequences alone did not distinguish between lice of different *Microcebus* species, possibly due to rather recent divergence. As bootstrap support for basal tree structure was rather low, further data are necessary to resolve the evolutionary history of louse-mouse lemur associations. Three new species of sucking lice are described: ***Lemurpediculus zimmermanni* sp. Nov.** From *Microcebus ravelobensis*, ***Lemurpediculus gerpi* sp.nov.** from *Microcebus gerpi*, and ***Lemurpediculus tsimanampesotsae* sp. nov.** from *Microcebus griseorufus*. These new species are compared with all known congeneric species and identifying features are illustrated for all known species of *Lemurpediculus*.

## Highlights

• Sucking lice (*Lemurpediculus* spp.) from mouse and dwarf lemurs were studied.
• Louse phylogenies were created based on three loci (COI, EF1α, ITS1).
• Results suggest a high host specificity of sucking lice from mouse and dwarf lemurs.
• Three new *Lemurpediculus* species are described.

## Graphical abstract

**Figure:**
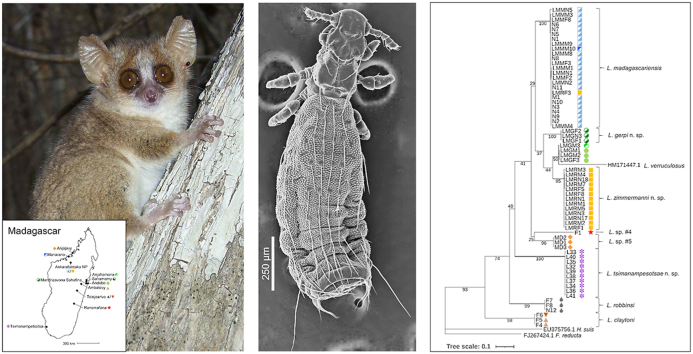

## 1 Introduction

Parasitic organisms constitute a major part of global biodiversity (Brooks and Hoberg, 2000; Whiteman and Parker, 2005). Sucking lice (Insecta: Phthiraptera: Anoplura) are obligate ectoparasites adapted to permanent life on their host and are generally characterized by a high degree of host specificity (Durden and Musser, 1994). Primate sucking lice, in particular, are considered to have mostly co-speciated with their hosts (Reed et al., 2007; Light and Reed, 2009). However, extant parasite-host associations may also result from host-switching, parasite duplication or parasite extinction events (Paterson and Gray, 1997; du Toit et al., 2013; Bell et al., 2021), and may thus generate or confirm hypotheses regarding historical host distribution/host dispersal (Johnson et al., 2021) or even changes in host behaviour (Kittler et al., 2003).

The island of Madagascar is characterized by an extraordinary level of biodiversity and species endemism, as exemplified by the primate genus *Microcebus*, the mouse lemurs, within the family Cheirogaleidae. More than twenty *Microcebus* species have been described to date, and genetic data suggest the existence of further, cryptic taxa (Hotaling et al., 2016), although the validity of some species is currently under debate (Poelstra et al., 2020). These small-bodied, nocturnal primates are found in forest habitats all over the island. Most species are microendemic to certain areas, often defined by natural barriers such as rivers (Olivieri et al., 2007; Kamilar et al., 2016; Schüßler et al., 2020). A few species, like the grey mouse lemur (*Microcebus murinus*), exhibit larger geographical distributions (Schneider et al., 2010; Blair et al., 2014), and may occur sympatrically with other microendemic species (e.g. Schmid and Kappeler, 1994; Zimmermann et al., 1998).

In contrast to the known diversity of cheirogaleid lemurs, only few of their louse species have been described: two from mouse lemurs – *Lemurpediculus verruculosus* from *Microcebus rufus* and *Lemurpediculus madagascariensis* from *M. murinus* – and two from the related genus *Cheirogaleus* (dwarf lemurs) – *Lemurpediculus claytoni* from *Cheirogaleus sibreei*, and *Lemurpediculus robbinsi* from *C. crossleyi* (Ward, 1951; Durden et al., 2010, 2017, 2018). Due to the high degree of host specificity among Anoplura, a number of undescribed louse species can thus be expected. However, exceptions regarding host specificity may occur, especially when considering congeneric hosts. For example, the sucking louse *Polyplax serrata* parasitizes several species of *Apodemus* mice (Stefka and Hypsa, 2008) and, similarly, different genotypes of morphologically indistinguishable *Polyplax arvicanthis* occur on different *Rhabdomys* mice which were formerly all considered to be *Rhabdomys pumilio* (du Toit et al., 2013). *Rhabdomys* mice have diverged relatively recently and their ranges are characterized by some degree of overlap, probably facilitating gene flow between their louse populations (du Toit et al., 2013). In contrast, restriction of host movement due to specific (micro-)habitat requirements may result in limited body contact between sympatric hosts and thus enhance host specificity (Bothma et al., 2019).

**Fig. 1:**
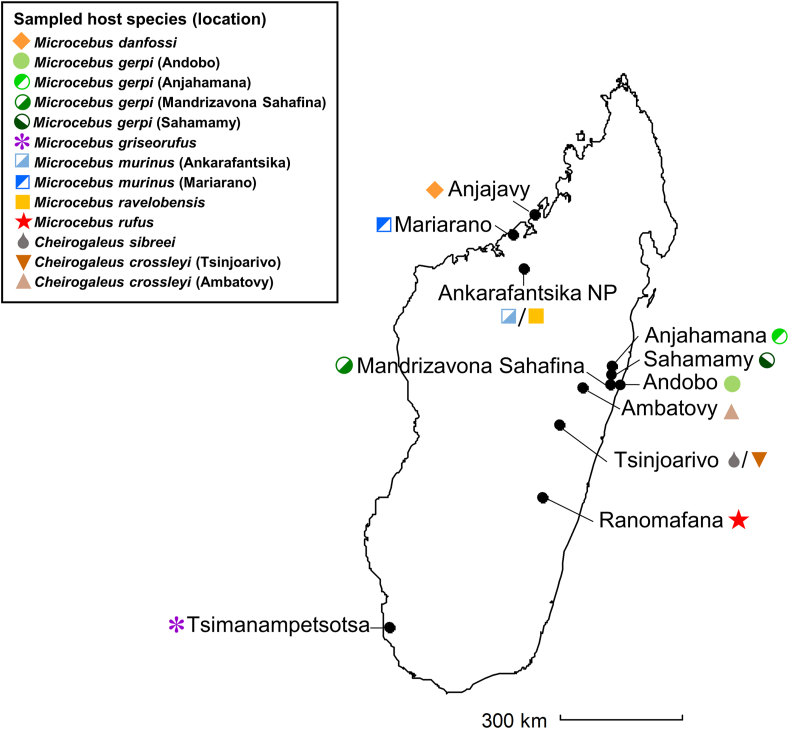
Sampling sites where cheirogaleids were trapped for collection of lice. Different colours/symbols correspond to the sampled host species/populations, with different shades of blue and green indicating different populations of *M. murinus* and *M. gerpi*, respectively. (For interpretation of the references to colour in this figure legend, the reader is referred to the Web version of this article.)

To shed more light on the species diversity and host specificity of lice parasitizing cheirogaleid lemurs, the present study analysed lice from six different *Microcebus* spp. and two *Cheirogaleus* spp. at ten sites in Madagascar, covering several different eco zones of the island (Fig. 1). Collected lice were characterized based on three different molecular markers: elongation factor 1α (EF1α) and cytochrome C oxidase subunit I (COI), which have been widely used in phylogenetic studies of lice (e.g. Johnson et al., 2002; Stefka and Hypsa, 2008; Light and Reed, 2009), as well as the internal transcribed spacer 1 (ITS1), which is frequently used in phylogenetic studies of other ectoparasites and often presents a greater degree of intra- and interspecific variation (Vobis et al., 2004, 2004; Øines and Brännström, 2011; de la Fuente et al., 2021). Adult specimens, if available, were additionally characterized by light and electron microscopy, resulting in the formal description of three new species. Based on molecular analyses, lice from two more species of mouse lemurs appear to represent two more undescribed species of *Lemurpediculus* but are not described here because adult louse specimens were not available.

## 2 Materials and methods

### 2.1 Louse sampling

Sucking lice were collected from six *Microcebus* spp. and two *Cheirogaleus* spp. during routine live-trapping procedures at ten locations in Madagascar (Table 1, Fig. 1) and stored in individually labelled vials in 70–90% ethanol. At two sites each, two sympatric host species were sampled (*M. murinus* and *M. ravelobensis* at Ankarafantsika National Park and *C. sibreei* and *C. crossleyi* at Tsinjoarivo), while lice from *Microcebus gerpi* were collected from three different, geographically separated, populations. Trapping procedures have been described by Durden et al. (2018), Klein et al. (2018) and Schüßler et al. (2020).

**Table 1:** Origin and number of sucking lice included in the phylogenetic study.

Host species	Sampling location	GPS coordinates	No. of lice
*Microcebus danfossi*	Anjajavy	−15.01 S, 47.24 E	3
*Microcebus gerpi*	Mandriza Sahafina	−18.81 S, 48.98 E	5
*Microcebus gerpi*	Andobo	−18.90 S, 49.13 E	3
*Microcebus gerpi*	Anjahamana	−18.39 S, 49.00 E	1
*Microcebus griseorufus*	Tsimanampetsotsa National Park	−24.02 S, 43.74 E	10
*Microcebus murinus*	Ankarafantsika National Park	−16.32 S, 46.72 E	24
*Microcebus murinus*	Mariarano	−15.48 S, 46.69 E	1
*Microcebus ravelobensis*	Ankarafantsika National Park	−16.32 S, 46.72 E	17
*Microcebus rufus*	Ranomafana National Park	−21.25 S, 47.42 E	3
*Cheirogaleus crossleyi*	Ambatovy	−18.87 S, 48.31 E	2
*Cheirogaleus crossleyi*	Tsinjoarivo	−19.69 S, 47.78 E	1
*Cheirogaleus sibreei*	Tsinjoarivo	−19.69 S, 47.78 E	3

### 2.2 Molecular and phylogenetic analyses

For DNA isolation, individual louse specimens were homogenized in 45 μl DirectPCR® Reagent (Cell) (PEQLAB Biotechnology GmbH, Erlangen, Germany) with the addition of 5 μl proteinase K, and incubated at 56 °C for 16 h, followed by 45 min at 85 °C for proteinase K inactivation.

Primers and thermocycling conditions used for PCR amplification of partial elongation factor 1α (EF1α), cytochrome C oxidase subunit I (COI) and internal transcribed spacer 1 (ITS1) regions are listed in Table 2. Each 50 μl PCR reaction included 0.5 μl DreamTaq® DNA polymerase (Thermo Fisher Scientific, Epsom, UK), 5 μl 10x buffer, 1 μl dNTPs (10 mM, Roti®-Mix PCR 3, Carl Roth GmbH + Co. KG, Karlsruhe, Germany) and 1 μl of each primer (10 μM each). The initial amount of template was 5 μl for EF1α and COI and 3 μl for ITS1. If no amplicon was observed after the first PCR run, the amount of template was increased to 10 μl (EF1α and COI) and 5 μl (ITS1), respectively. Amplicons were visualized by electrophoresis on 1.5% agarose gels stained with GelRed® (Biotium Inc., Fremont, California, USA). In a few cases, multiple bands were observed and bands of the expected size were excised from the gel and centrifuged for 2 min at 2300×*g* through a pipette filter tip. One microliter of the resulting filtrate was subjected to reamplification as described above.

**Table 2:** Primers and thermocycling conditions used for amplification of louse DNA.

Gene/region	Primer name	Primer sequence (5′-3′)	Primer reference	Thermoprofile
EF1α	EF-For3	GGN GAC AAY GTT GGY TTC AAC G	Danforth and Ji (1998)	95 °C 1 min; 5 × 95 °C 30 s, 48 °C 30 s, 72 °C 1 min; 35 × 95 °C 30 s, 52 °C 30 s, 72 °C 1 min; 72 °C 10 min
	CHo10	ACRGCVACKGTYTGHCKCATGTC		
COI	L6625	CCGGATCCTTYTGRTTYTTYGGNCAYCC	Hafner et al. (1994)	95 °C 1 min; 5 × 95 °C 30 s, 48 °C 30 s, 72 °C 1 min; 30 × 95 °C 30 s, 55 °C 30 s, 72 °C 1 min; 72 °C 10 min
	H7005	CCGGATCCACNACRTARTANGTRTCRTG		
ITS1	CAS18sF1	TACACACCGCCCGTCGCTACTA	Ji et al. (2003)	95 °C 1 min; 38 × 95 °C 30 s, 62 °C 30 s, 72 °C 1 min; 72 °C 10 min
	L18sFn	GGTCTTTGGACTCGTACGCG	This study	

Amplicons were custom Sanger sequenced in both directions (MicroSynth Seqlab, Göttingen, Germany). After quality control, consensus sequences were assembled and alignments created with Clone Manager Professional v. 9 (Sci Ed Software, Westminster, Colorado, USA). Alignments were trimmed to 340 (EF1α), 349 (COI) and 692 (ITS1) nucleotide sites, respectively. P-distances were calculated in MEGA v. 11 (Tamura et al., 2021). Maximum likelihood phylogenetic trees were computed via the IQ-TREE web server (Trifinopoulos et al., 2016), which employs ModelFinder to identify the best-fit substitution model according to the Bayesian information criterion (BIC) (Kalyaanamoorthy et al., 2017). Ultrafast bootstrap with 1000 replicates was used to estimate branch support values (Hoang et al., 2017). Trees were constructed for each of the three genes separately, as well as for the concatenated sequences. For the latter, an edge-linked proportional partition model (Chernomor et al., 2016) was employed, which showed a better fit based on log-likelihood, BIC and Akaike information criterion (AIC) values as compared to a non-partitioned model. Publicly available EF1α and COI sequences of *L. verruculosus* (Genbank accession nos. HM171447 and HM171479) were included, and sequences of *Fahrenholzia* spp. and *Haematopinus suis* were used as outgroups, except for ITS1, for which no Phthiraptera sequences were publicly available at the time of analysis. Trees were visualized using Interactive Tree Of Life v. 6 (Letunic and Bork, 2021).

### 2.3 Morphological analyses

Samples of sucking lice stored in vials containing 70–90% ethanol were prepared for light microscopy as type or voucher specimens. These specimens were cleared in 10% potassium hydroxide, rinsed in distilled water, dehydrated through an ethanol series, further cleared in xylene, slide-mounted in Canada balsam and then oven-dried for two weeks. Line drawings were prepared by examining specimens at 100–400x with an Olympus BH-2 phase contrast high-power microscope (Olympus Corporation of the Americas, Center Valley, Pennsylvania) connected to an Ikegami MTV-3 video camera attachment and monitor (Ikegami Electronics, Neuss, Germany). Measurements were made using a calibrated ocular micrometer. Line drawings of diagnostic characters for previously described species of *Lemurpediculus* were made from slide-mounted specimens in the collection of L. A. Durden. Standardized descriptive format for Anoplura and morphological terminology follow Kim and Ludwig (1978) and Durden et al. (2018). Names of setae are spelled out at first mention (with acronyms in parentheses) followed by use of acronyms subsequently. Important structures are labelled in plates. Images were stitched together using the “Photomerge” automation application in Adobe Photoshop Creative Cloud (2018)(Adobe Inc., San Jose, California, USA). Stacked microphotographic images of slide-mounted whole specimens of both sexes for each new species of louse were prepared using a Visionary Digital K2/SC long-distance microscope (Infinity Photo-Optical Company, Boulder, Colorado, USA).

Two male and two female lice collected from *M. ravelobensis* were prepared for Scanning Electron Microscopy (SEM) (one of each sex mounted dorsally and one of each sex mounted ventrally) to show morphological characters of *Lemurpediculus* lice that are not readily visible by light microscopy such as the large posteriorly-directed ventral spikes on the head of the three new species described in this paper. Specimens were prepared for SEM as follows. Lice were dehydrated in a graded ethanol series; 70% for 30 min, 80% for 30 min, 90% for 1 h, 95% overnight, and 100% for 24 h. Following dehydration, specimens were chemically dried using a graded ethanol/hexamethyldisilazane (HMDS) series; 2(EtOH):1(HMDS) for 2 h, 1:1 for 2 h, 1:2 for 3 h, 1:3 overnight, pure HMDS for 6 h, followed by replacement of old HMDS with new pure HMDS, and allowed to evaporate overnight. Dried specimens were mounted on an aluminum stub, sputter coated with gold/palladium, and visualized on a JEOL JSM6610LV SEM (JEOL USA, Peabody, Massachusetts) at 15 KV. Multiple images were captured and stitched together using the photomerge function in Adobe Photoshop CC.

## 3 Results

### 3.1 Phylogenetic analyses

A total of 73 lice of the genus *Lemurpediculus* were included in the phylogenetic analyses (Table 1). PCR amplification and sequencing success varied between the three genes. High quality sequences of partial EF1α (GenBank accession nos. OP133931– OP133998) and COI (accession nos. OP078637-OP078701) were generated for 68 and 65 lice, respectively, whereas sequencing of ITS1 amplicons proved difficult in many cases due to the presence of a poly-T motif, resulting in only 40 successfully sequenced lice (accession nos. OP115441-OP115480; Table S1). Sequences for all three loci were generated for 36 lice.

The three loci differed in their degree of phylogenetic resolution. The 340 bp-EF1α sequences of the lemur lice showed the lowest degree of variation (mean uncorrected p-distances: 1.6% [range: 0.0–11.3%]) and the dataset underlying the EF1α phylogenetic tree (including outgroups) contained only 60 potentially parsimony informative sites. Greater variation was observed for COI (mean uncorrected p-distances of 13.8% [0.0–25.8%], 142/349 sites potentially parsimony informative) and ITS1 (mean uncorrected p-distances of 5.1% [0.0–25.4%], 142/692 sites potentially parsimony informative, including the flanking regions of the 18 S and 5.8 S rDNA).

**Fig. 2:**
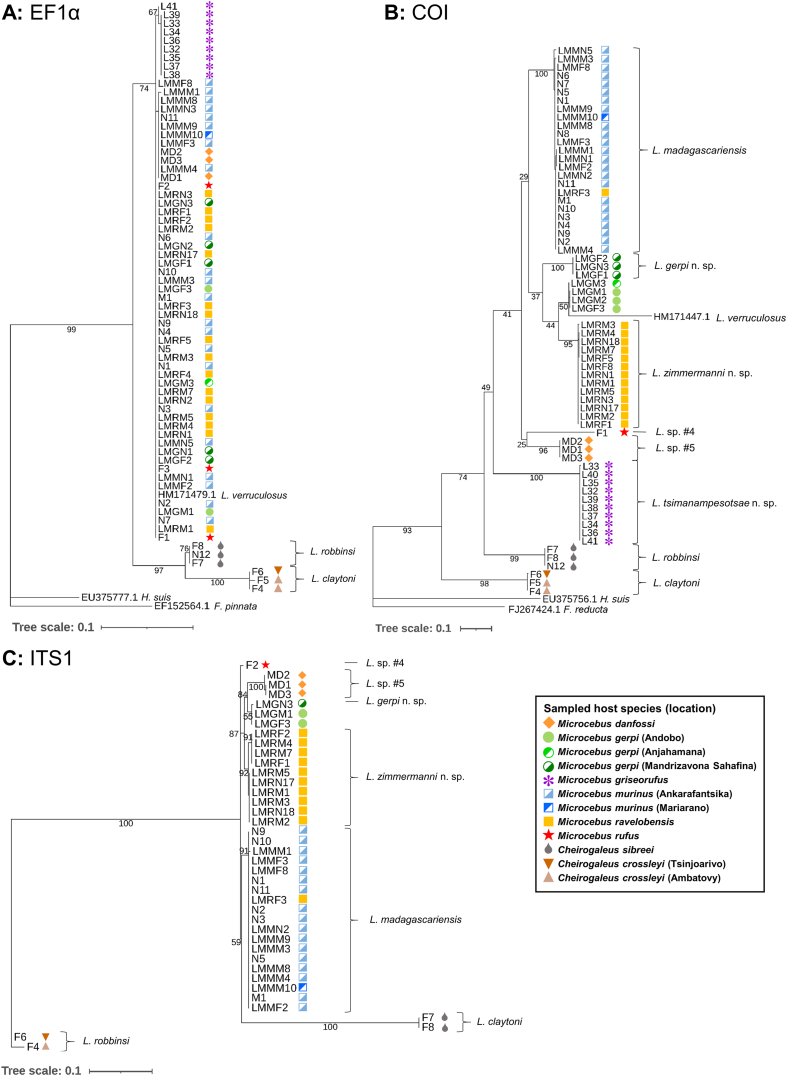
Maximum likelihood phylogenetic trees based on A) elongation factor 1α (EF1α), B) cytochrome C oxidase subunit I (COI) and C) internal transcribed spacer 1 (ITS1) sequences. Values next to the branches indicate ultrafast bootstrap support. Tree scale is in substitutions/site. Substitution models were TNe + G4 for EF1α, TPM2+F + I + G4 for COI, and F81 + F + I for ITS1. Abbreviations: *F.*, *Fahrenholzia*; *H., Haematopinus*; *L.*, *Lemurpediculus*.

**Fig. 3:**
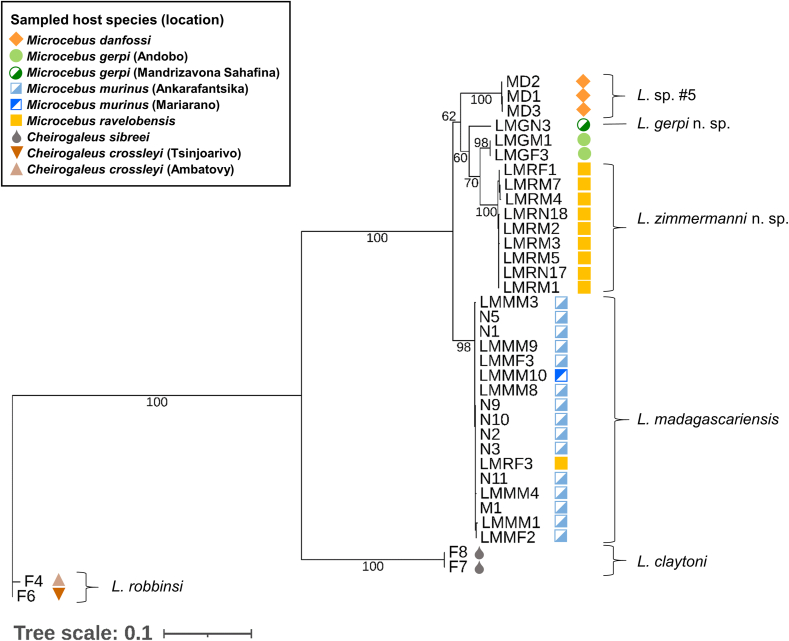
Maximum likelihood phylogenetic tree based on concatenated cytochrome C oxidase subunit I (COI), elongation factor 1α (EF1α) and internal transcribed spacer 1 (ITS1) sequences. Substitution models were TPM2u + F + I + G4 for COI, TIM2e for EF1α and F81 + F + I for ITS1. Values next to the branches indicate ultrafast bootstrap support. Tree scale is in substitutions/site. Abbreviations: *L.*, *Lemurpediculus*.

In the maximum likelihood tree based on EF1α sequences (Fig. 2A), all lice from the six different *Microcebus* spp. hosts formed a single cluster without any inner resolution. In fact, all of these sequences showed at least 98.0% nucleotide identity. In contrast, COI and ITS1 sequences generally clustered by host species (Fig. 2A and C), with the exception of a single louse specimen labelled as originating from *M. ravelobensis,* which clustered with specimens from *M. murinus.* However, it cannot be excluded that this specimen was mislabelled or that the host was misidentified. The clade of lice collected from *M. murinus* corresponds to *L. madagascariensis* (Durden et al., 2018), and those from *Cheirogaleus sibreei* and *C. crossleyi* to *L. claytoni* and *L. robbinsi,* respectively (Durden et al., 2017), while the remaining five louse clades cannot be assigned to any known species. Three of these are formally described as new species below (*Lemurpediculus zimmermanni* sp. nov., *L. gerpi* sp. nov., and *L. tsimanampesotsae* sp. nov.), while the remaining two candidate species are labelled *Lemurpediculus* sp. nov. #4 and #5 in Fig. 2, Fig. 3. Interestingly, the COI sequence generated from a louse specimen collected from *M. rufus* in the present study did not cluster with previously published sequences of *L. verruculosus* from this host species (Fig. 2B). Unfortunately, no published ITS1 sequence is yet available for comparison.

Regarding lice from *Microcebus gerpi* (*L. gerpi* sp. nov.), the COI tree suggested paraphyletic phylogenetic structure of lice sampled from different host populations. However, the cluster of sequences from the Andobo and Anjahamana populations showed low bootstrap support (Fig. 2B). In the ITS1 tree, on the other hand, the sequences from the three sampled populations clustered together. Nevertheless, we exercise caution due to these ambiguous molecular results and only describe *L. gerpi* based on specimens (both males) from one site (Mandrizavona Sahafina) despite the morphological congruence with other male *Lemurpediculus* specimens from *M. gerpi* at the other two sites.

While most terminal nodes were well supported in the COI tree (bootstrap support >95%), most basal nodes showed rather low bootstrap support (Fig. 2B). Regarding ITS1, bootstrap support was generally high (>80%), with few exceptions (Fig. 2C). The COI and ITS1 trees showed some topological differences, with *L. gerpi* sp. nov. Appearing as a sister taxon to *L. zimmermanni* sp. nov. In the COI tree, but to *Lemurpediculus* sp. nov. #5 in the ITS1 tree. Moreover, *L. claytoni* appeared to be monophyletic with the lice of *Microcebus* spp. in the ITS1 tree, in contrast to the COI- and EF1α-based phylogenies.

### 3.2 Descriptions of the new species

Polyplacidae Fahrenholz, 1912.

*Lemurpediculus* Paulian, 1958.

Type species: *Lemurpediculus petterorum* Paulian, 1958.

#### *3.2.1* *Lemurpediculus zimmermanni* n. sp. Durden, Springer, Kiene, Klein, Rakotondravony, Ehlers, Greiman, Blanco, Zohdy, Kessler, Strube & Radespiel

**Fig. 4:**
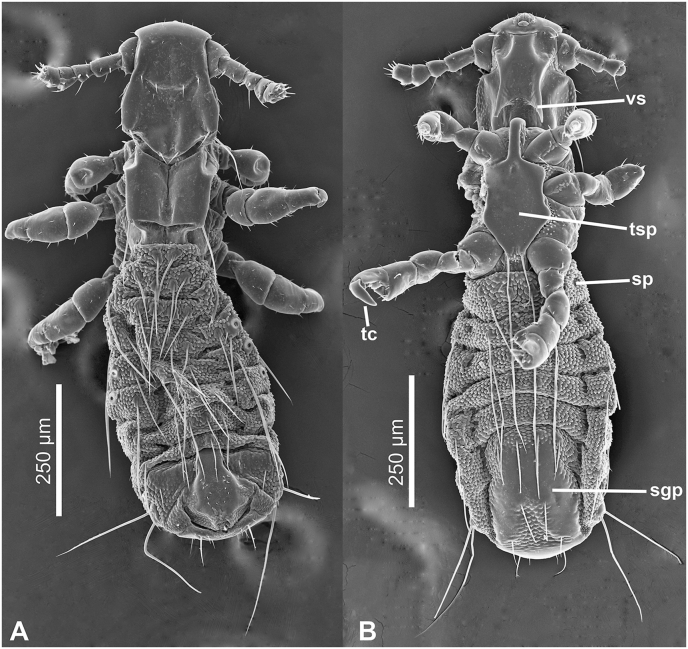
***Lemurpediculus zimmermanni,*** male, scanning electron micrographs: A, dorsal whole body. B, ventral whole body. **Abbreviations sgp**, subgenital plate; **sp**, spiracle; **tc**, tibiotarsal claw; **tsp**, thoracic sternal plate; **vs**, ventral head spike.

**Fig. 5:**
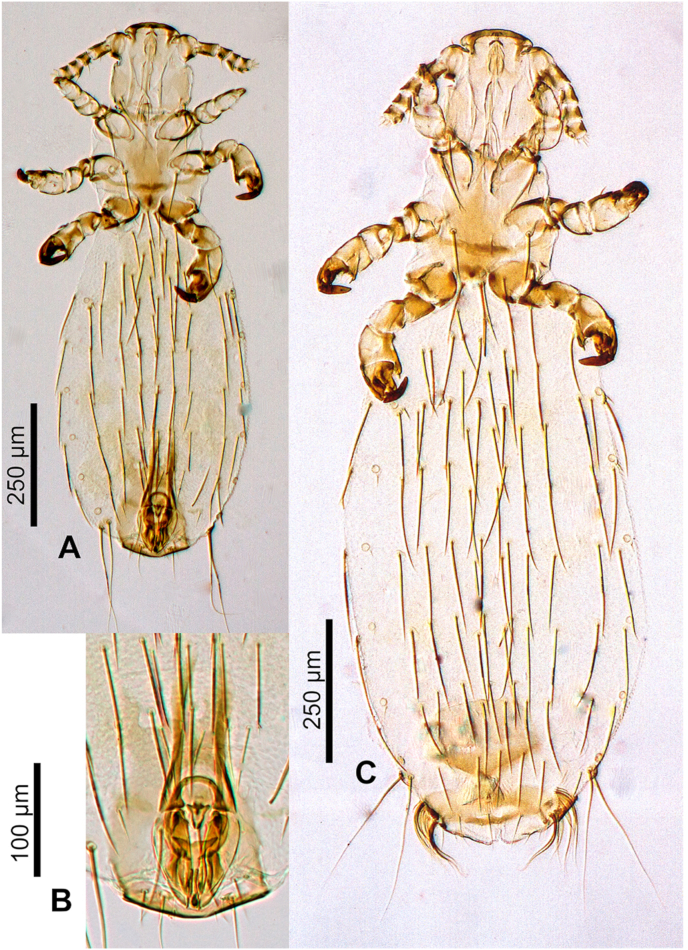
***Lemurpediculus zimmermanni,*** stacked photoimages: A, male Holotype, USNMENT00981950, whole body. B, male, Holotype, USNMENT00981950, genitalia. C, female Allotype, USNMENT00981951, whole body.

**Fig. 6:**
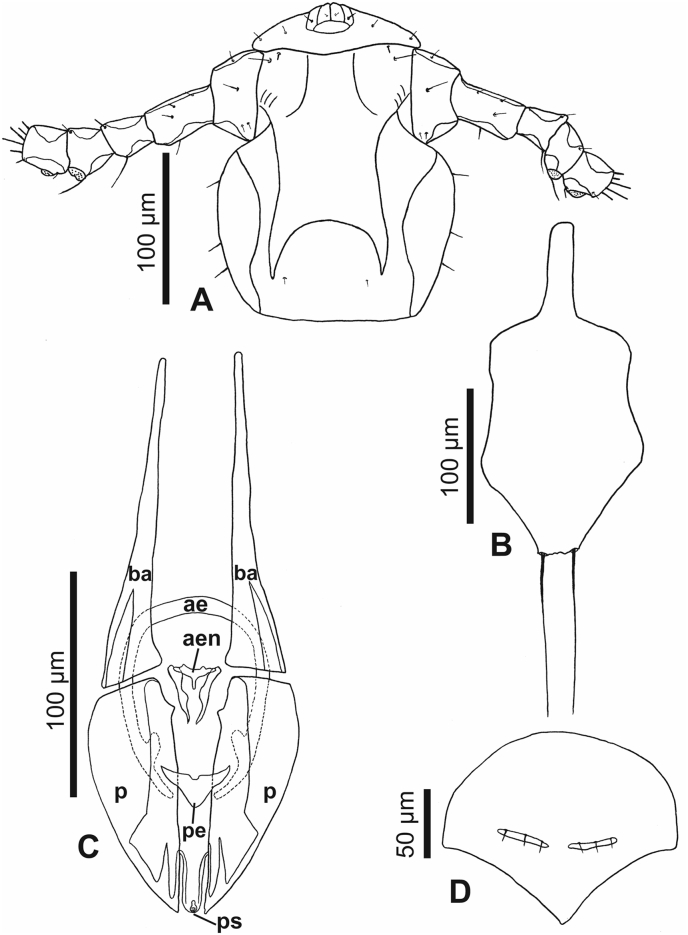
***Lemurpediculus zimmermanni,*** A: Ventral head of male Holotype, B: Thoracic sternal plate of Holotype male, C: Genitalia of male Holotype, D: Subgenital plate of female allotype. **Abbreviations**: **ae**, anterior endomere; **aen**, aedeagal endomere; **ba**, basal apodeme; **p**, paramere; **pe**, posterior endomere; **ps**, pseudopenis.

**Fig. 7:**
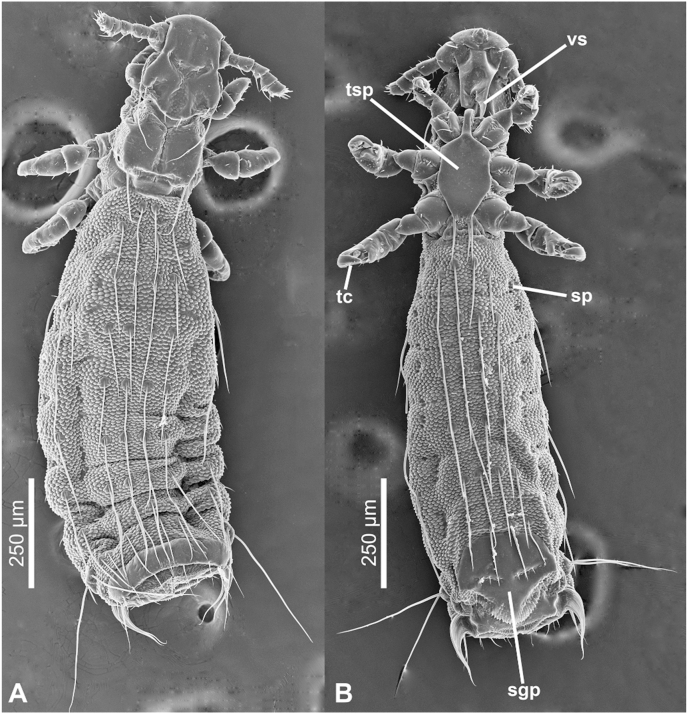
***Lemurpediculus zimmermanni,*** female, scanning electron micrographs: A, dorsal whole body. B, ventral whole body. **Abbreviations: sgp**, subgenital plate; **sp**, spiracle; **tc**, tibiotarsal claw; **tsp**, thoracic sternal plate; **vs**, ventral head spike.

Adult male and female (Fig. 4, Fig. 5, Fig. 6, Fig. 7).

***Material studied.*** 3 adult males, 6 adult females.

**Description:**

*MALE (*Fig. 4, Fig. 5, Fig. 6A,B,C*) (n* = *3):* Total body length of Holotype, 1.070 mm (mean, 1.028 mm; range, 0.992–1.070 mm). Head and thorax moderately sclerotized; abdomen weakly sclerotized.

*Head (*Fig. 4, Fig. 5, Fig. 6A*):* Longer than wide with smoothly rounded, sclerotized anterior margin, indented anterio-lateral dorsal margins, broadening posteriorly, and then indented and narrowing to rounded posterior margin dorsally. Massive and distinctive protuberance on ventral head bearing two large posterolaterally-directed acuminate (pointed) spikes. Head with smooth integument. Maximum head width of Holotype, 0.162 mm (mean, 0.160 mm, range, 0.148–0.170 mm). One central Principal Head Seta, 1 small Ventral Preantennal Seta, and 3 Oral Setae on each side. Two Sutural Head Setae, 3 Dorsal Marginal Head Setae, 3–4 Anterior Marginal Head Setae, 1 Dorsal Anterior Head Seta, 1 Dorsal Anterior Central Head Seta, 1 stout, medially-directed Dorsal Posterior Central Head Seta, 1 long Dorsal Principal Head Seta, and 1 stout Dorsal Accessory Head Seta (borne on small protuberance) on each side. Antennae 5-segmented; segment 1 much broader than long, segment 2 much longer than broad, segments 3 and 4 about as broad as long and broadening apically, segment 5 about as broad as long and not broadening apically; patches of sensilla present postero-apically on segments 4 and 5 in addition to terminal sensilla on segment 5. No distinct sexual dimorphism in antennal segments.

*Thorax (*Fig. 4, Fig. 6B*):* Slightly broader than head; maximum thorax width of Holotype, 0.235 mm (mean, 0.225 mm; range, 0.215–0.235 mm). Thoracic sternal plate (Fig. 4, Fig. 6B) with elongate narrow anterior process and small anterolateral and posterolateral rounded protuberances on main body of plate which narrows posteriorly to indented posterior margin; 2 long posteriorly-directed setae inserted on posterior margin of plate. Dorsal thorax with distinctive large squarish sclerotized plate with anteromedial indentation, anterolateral indentation, median depression with notal pit, and small posterior rounded extension. Thorax with smooth integument. Mesothoracic spiracle diameter of Holotype, 0.023 mm (mean, 0.023 mm; all specimens, 0.023 mm). One Dorsal Principal Thoracic Seta (DPTS) on each side but no accessory setae present. DPTS length in Holotype, 0.117 mm (mean, 0.109 mm, 0.105–0.117 mm). Legs each terminating in broad tibio-tarsal claw; claws slightly increasing in size from forelegs to midlegs and hindlegs; coxae variously shaped (Fig. 4B).

*Abdomen (*Fig. 4, Fig. 5A*):* Broader than thorax with mammillated integument except on subgenital plate (Fig. 4, Fig. 5A). Tergites and Sternites absent (as for genus). Two long Dorsal Central Abdominal Setae (DCAS) on abdominal segment 1, 4 DCAS on segments 2–8, 1 long Dorsal Lateral Abdominal Seta (DLAS) on each side on segments 4–6 and 8; 1 short medial seta and 1 long lateral seta on each side of subgenital plate. Two long setae and 4–5 shorter setae at posterior margin of abdomen. Four long Ventral Central Abdominal Setae (VCAS) on each of abdominal segments 2–7, 2 long DCAS on segment 9 and 2 shorter DCAS on segment 10. All DCAS, DLAS, VCAS and VLAS borne on small sclerites. Pair of lateral spiracles present on abdominal segments 3–8. Small seta posterior to penultimate spiracle and pair of long marginal setae posterior to last spiracle on each side. Small subtriangular paratergal plate present on each side of abdominal segment 4, and surrounding spiracle on that segment; with 1 long internal Paratergal Seta (PrS) and 1 short internal PrS on paratergal plate.

*Genitalia (*Fig. 5, Fig. 6C*):* Basal apodeme distinctly longer than parameres and differentially sclerotized, posteriorly. Parameres very broad with truncate anterior margin and tapering posteriorly to acuminate apex; smoothly curved lateral margins and straight medial margins except for antero-medial notch on each paramere; distinct posterior bifidly sclerotized areas on each paramere. Anterior subcircular endomere curving posteriorly to converging arms each with rounded apex; medial rounded lobe on each converging arm of anterior endomere just posterior to medial notch on each paramere. V-shaped posterior endomere, with small anterior indentation, situated between posteriorly extending arms of anterior endomere. Broad aedeagal sclerite with elongate posterior extension, situated between anterior and posterior endomeres, at junction of basal apodeme and parameres; elongate, sinuous sclerite present on both sides of aedeagal sclerite. Pseudopenis narrow, smoothly rounded apically, and just extending to apices of parameres. Subgenital plate (Fig. 4, Fig. 5B) distinctly smooth in contrast to surrounding mammillate abdominal integument, extending anteriorly to abdominal segment 6, with 2 broad posterior lobes, slightly tapering anteriorly to 2 anterolateral extensions. Dorsal outline of subgenital plate also smooth and with 20–22 small setae.

*FEMALE (*Fig. 5, Fig. 6, Fig. 7A,B*) (n* = *6):* Total body length of Allotype, 1.425 mm (mean, 1.343 mm; range, 1.153–1.425 mm). Head, thorax and abdomen as in male unless indicated otherwise.

*Head (*Fig. 5, Fig. 7A,B*):* Maximum head width of Allotype, 0.180 mm (mean, 0.172 mm; range, 0.164–0.180 mm).

*Thorax (*Fig. 5, Fig. 7A,B*):* Maximum thorax width of Allotype, 0.245 mm (mean, 0.234 mm; range, 0.223–0.251 mm). Mesothoracic spiracle diameter of Allotype, 0.024 mm (mean, 0.024 mm; range, 0.023–0.024 mm). DPTS length of Allotype, 0.122 mm (mean, 0.114 mm; range, 0.111–0.122 mm). Two long posterior setae present on thoracic sternal plate on 1 side in 1 specimen.

*Abdomen (*Fig. 5, Fig. 7A,B*):* Broader than thorax. Nine rows of long DCAS; row 1 with 2 DCAS, rows 2–9 each with 4 DCAS. Smoothly curved plate posterior to last row of DCAS with 7–8 slightly shorter setae. One long DMAS on each side of abdominal segments 3–8. Eight rows of VCAS; row 1 with 2 VCAS, rows 2–8 each with 4 VCAS. One long VMAS on each side of abdominal segments 3–7.1 small seta posterior to each spiracle.

*Genitalia (*Fig. 5, Fig. 6, Fig. 7B*):* Subgenital plate surface smooth, broadly curved anteriorly, then indented in posterior portion and tapering posteriorly to acuminate apex. Two horizontally elongate lacunae in plate, one on each side of midline; each lacuna with 3 short setae. Vulvar fimbriae extensive. Gonopods indistinct; gonopods VIII each with 3 small setae; gonopods IX lacking setae. Patch of ∼20 long, curved setae posterolateral to gonopods on each side. Small terminal setae (4–6) present on each side of genital aperture.

#### 3.2.2 Taxonomic summary

***Type host:****Microcebus ravelobensis*Zimmermann et al. (1998) (Golden-Brown Mouse Lemur).

***Type locality:*** Madagascar (northwestern): Boeny Region, Ankarafantsika National Park, Ambanjabe landscape (coordinates: −16.32 S, 46.72 E), collector: Frederik Kiene, August 2017.

**Site of infestation:** External body surface and fur.

***Type specimens:*** Holotype male (USNMENT00981950), Allotype female (USNMENT00981951), 1 Paratype male, 4 Paratype females; U.S. National Museum of Natural History, Smithsonian Institution, Washington DC, USA.

***Etymology:*** This species is named for the late Prof. Dr. Elke Zimmermann in recognition of her long-term engagement for the Ankarafantsika National Park and its lemur fauna, her major scientific achievements, and her never-ending interest in mouse lemur biology, socio-ecology, communication, evolution and health. In describing the host species (*M. ravelobensis*) of *L. zimmermanni* in 1998 (Zimmermann et al., 1998), she also facilitated a better understanding of the native parasite diversity in Madagascar. ***Zoobank accession number:*** LSID:urn:lsid:zoobank.org.act: 4FEC9CCA-C72D-4BF3–8C02-D0388003E820.

#### *3.2.3* *Lemurpediculus gerpi* n. sp. Durden, Springer, Kiene, Klein, Rakotondravony, Ehlers, Greiman, Blanco, Zohdy, Kessler, Strube & Radespiel

**Fig. 8:**
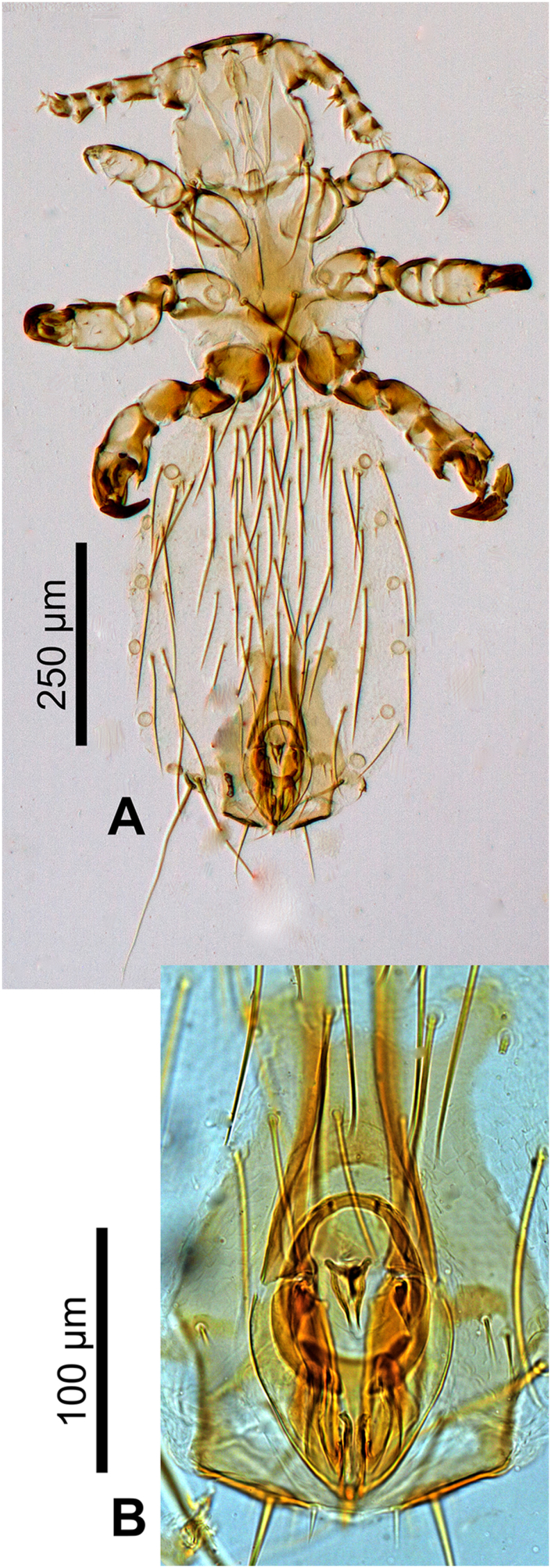
***Lemurpediculus gerpi,*** stacked photoimages: A, male Holotype, USNMENT00981952, whole body. B, male, Holotype, USNMENT00981952, genitalia.

**Fig. 9:**
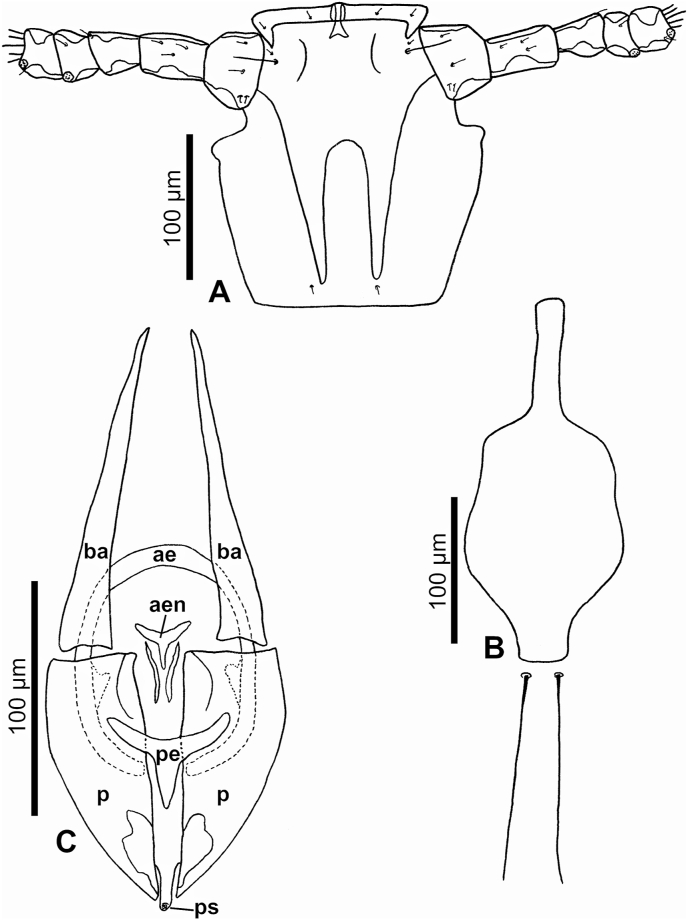
***Lemurpediculus gerpi,*** A: Ventral head of male Holotype, B: Thoracic sternal plate of Holotype male, C: Genitalia of male Holotype. **Abbreviations**: **ae**, anterior endomere; **aen**, aedeagal endomere; **ba**, basal apodeme; **p**, paramere; **pe**, posterior endomere; **ps**, pseudopenis.

Adult male (Fig. 8, Fig. 9).

***Material studied.*** 2 adult males.

***Description:***

*MALE (*Fig. 8, Fig. 9A,B,C*) (n* = *2):* Total body length of Holotype, 1.002 mm (mean, 1.028 mm; range, 1.002–1.053 mm). Morphology as for *L. zimmermanni* sp. nov. unless stated otherwise.

*Head (*Fig. 8, Fig. 9A*):* Longer than wide; anterior margin almost straight and heavily sclerotized. Lateral margins of head indented immediately posterior to antennae, then bulging laterally to small protuberance on each side, and then slightly tapering to straight posterior margin. Ventral surface of head with two massive narrow acuminate spikes; spikes distinctly longer and narrower than spikes on ventral head in *L. zimmermanni* sp. nov. or *L. tsimanampesotsae* sp. nov. Antennal morphology and head setae as in *L. zimmermanni* sp. nov. except antennal segment 5 slightly broader than long. Maximum head width of Holotype, 0.181 mm (mean, 0.175 mm; range, 0.168–0.181 mm).

*Thorax (*Fig. 8, Fig. 9B*):* Thorax slightly wider than head. Maximum thorax width, 0.255 mm (mean, 0.245 mm; range 0.234–0.255 mm). Thoracic sternal plate (Fig. 9B) similar to that of *L. zimmermanni* sp. nov. but posterior margin straight and 2 long setae inserted just posterior to posterior margin of plate. DPTS length of Holotype, 0.135 mm (mean, 0.130 mm: range, 0.125–0.135 mm). Mesothoracic spiracle diameter of Holotype, 0.025 mm (mean, 0.026 mm; range, 0.025–0.026 mm).

*Abdomen (*Fig. 8A*):* Broader than thorax with mammillated integument except on subgenital plate. Two long DCAS on each of abdominal segments 1 and 2; 4 long DCAS on each of segments 3–9; 2 long DCAS on segment 10. One long DLAS on each side on segments 3–7; 1 short medial seta and 1 long lateral seta on each side of subgenital plate. Two long setae and 2 shorter setae at posterior margin of abdomen. Four long VCAS on each of abdominal segments 2–8, and 4 shorter VCAS on segment 9. One long VLAS present on each side on segments 2–7. Pair of lateral spiracles present on abdominal segments 3–8. Pair of long marginal setae posterior to last spiracle on each side. Small subtriangular paratergal plate present on each side of abdominal segment 4, and surrounding spiracle on that segment; with 1 long PrS and 1 short PrS on paratergal plate.

*Genitalia (*Fig. 8, Fig. 9C*):* Basal apodeme slightly longer than parameres and with arms distinctly tapering and converging anteriorly. Parameres very broad anteriorly, tapering posteriorly to acuminate apex, and with straight medial margins and smoothly curved lateral margins; distinct posterior sclerotized areas on each paramere (different in shape to sclerotized areas in *L. zimmermanni* sp. nov. and *L. tsimanampesotsae* sp. nov.). Anterior subcircular endomere curving posteriorly to converging arms each with rounded apex and with small medial protrusion on each side near anterior margin of parameres. Y-shaped posterior endomere situated between posterior arms of subcircular endomere and smaller Y-shaped aedeagal endomere situated between junction of parameres and basal apodeme; aedeagal endomere with 1 vertically elongate postero-lateral sclerite on each side. Pseudopenis narrow and extending slightly beyond posterior apices of parameres. Subgenital plate (Fig. 8B) with curved posterior margin medially and straight posterior margins laterally, progressing to slightly concave lateral margins leading to small apex on each side, and converging concave lateral margins extending to anterolateral extension on each side.

#### 3.2.4 *Taxonomic summary*

***Type host:****Microcebus gerpi* Radespiel, Ratsimbazafy, Rasoloharijaona, Raveloson, Andriaholinirina, Rakotondravony, Randrianarison and Randrianambinina, 2011(Gerp's mouse lemur)

***Type locality:*** Madagascar (east-central): Tamatave Province, Mandrizavona Sahafina (−18.810 S, 48.976 E) (Holotype male and Paratype male), collector: Romule Rakotondravony, collection date: September 11, 2018.

**Site of infestation:** External body surface and fur.

***Type specimens:*** 1 male Holotype (USNMENT00981952), 1 male Paratype, U.S. National Museum of Natural History, Smithsonian Institution, Washington DC, USA.

***Etymology:*** This new species is named for its host, *Microcebus gerpi.*

***Zoobank accession number:* LSID:** urn:lsid:zoobank.org.act: 99D06D25-6DE4-4FBC-93F1-D59F13750602.

***Other material examined:*** We have also examined 1 male *Lemurpediculus* louse collected from *M. gerpi* at Andobo (18.904 S, 49.126 E) (collector: Romule Rakotondravony, Aug. 26, 2018) that morphologically matches the holotype male of *L. gerpi* from Sahafina but we do not include it in the species description because of the ambiguous molecular results documented in this paper. Further, we have examined 2 accompanying female *Lemurpediculus* lice from *M. gerpi* at Andobo (collector: Romule Rakotondravony, Aug. 18, 2018) that do not match females of any previously described lice in this genus, and another 3 morphologically identical females from *M. gerpi* from Sahamamy (18.564 S, 48.979 E) (collector: Romule Rakotondravony, Oct. 12, 2018). For the same reason cited above, we do not include any of these females in the description of *L. gerpi*.

#### *3.2.5* *Lemurpediculus tsimanampesotsae* n. sp. Durden, Springer, Kiene, Klein, Rakotondravony, Ehlers, Greiman, Blanco, Zohdy, Kessler, Strube & Radespiel

**Fig. 10:**
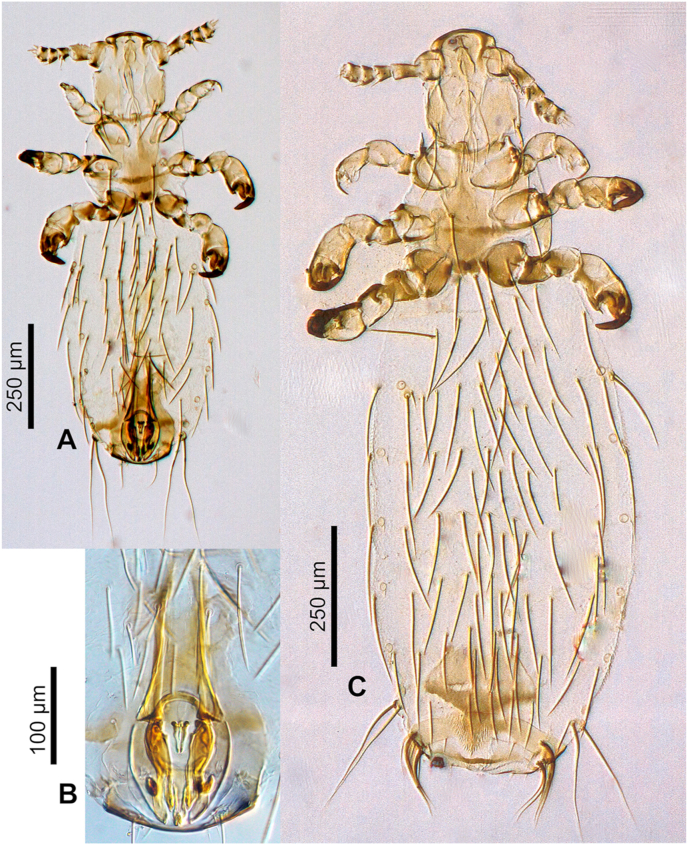
***Lemurpediculus tsinamanpesotsae,*** stacked photoimages: A, male Holotype, USNMENT00981954, whole body. B, male, Holotype, USNMENT00981954, genitalia. C, female Allotype, USNMENT00981955, whole body.

**Fig. 11:**
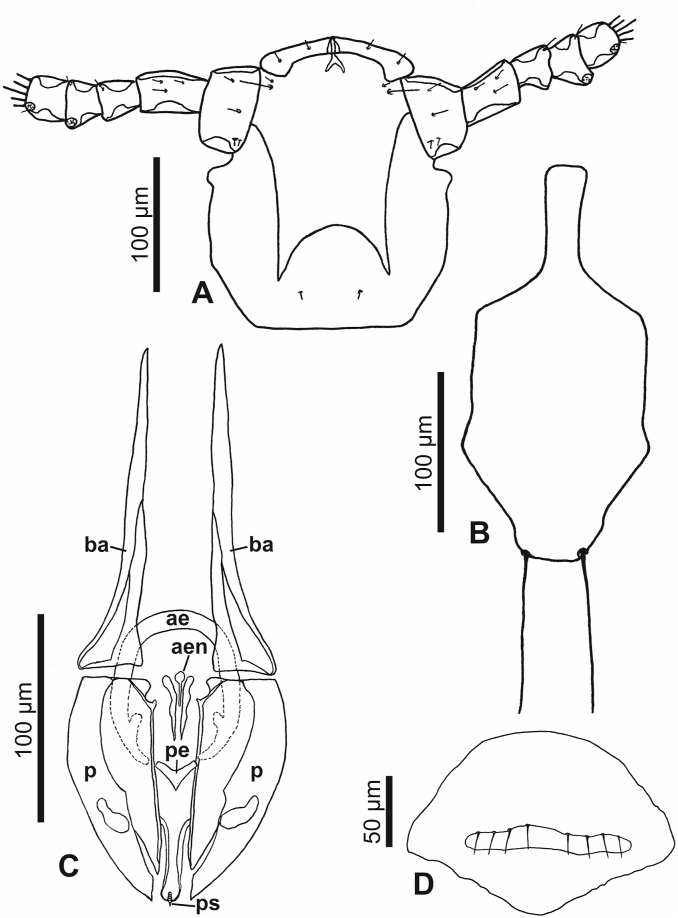
***Lemurpediculus tsinamanpesotsae,*** A: Ventral head of male Holotype, B: Thoracic sternal plate of Holotype male, C: Genitalia of male Holotype, D: Subgenital plate of female Allotype. **Abbreviations**: **ae**, anterior endomere; **aen**, aedeagal endomere; **ba**, basal apodeme; **p**, paramere; **pe**, posterior endomere; **ps**, pseudopenis.

Adult male and female (Fig. 10, Fig. 11).

***Material studied.*** 1 adult male, 1 adult female.

***Description:***

Note: This species was listed as *Lemurpediculus verruculosus* by Ehlers et al. (2019).

*MALE (*Fig. 10, Fig. 11A,B,C*) (n* = *1):* Total body length of Holotype, 1.051 mm. Morphology as in *L. zimmermanni* sp. nov. unless stated otherwise.

*Head (*Fig. 10, Fig. 11A*):* Rounded, sclerotized, anterior margin; lateral margins indented immediately posterior to antennae, then bulging as small protrusion on each side, followed by almost straight and parallel margins and tapering slightly to straight posterior margin. Massive protuberance on ventral head bearing 2 large spikes (but shorter than in *L. zimmermanni* sp. nov. and *L. gerpi* sp. nov.); margin between spikes semicircular. Remaining head morphology and setation as for *L. zimmermanni* sp. nov. Maximum head width of Holotype, 0.185 mm.

*Thorax (*Fig. 10, Fig. 11B*):* Thoracic sternal plate similar to that of *L. zimmermanni* sp. nov. but posterior margin slightly rounded and pair of posterior setae shorter. Maximum width of thorax in Holotype, 0.250 mm. DPTS length of Holotype, 0.106 mm. Mesothoracic spiracle diameter of Holotype, 0.023 mm.

*Abdomen (*Fig. 10A*):* Eight rows of long DCAS; row 1 with 2 setae, rows 2–8 each with 4 setae. One long DLAS on each side on segments 2–6. Six rows of long VCAS; rows 1–5 each with 4 setae; row 6 with 2 setae. One long VLAS on each of segments 3–6. Anterior spiracle with small subtriangular paratergal plate with 1 long and 1 short PrS. Two long lateral setae on each side on segment 8. Abdominal apex with 1 long apical seta and 5–6 small setae on each side.

*Genitalia (*Fig. 10, Fig. 11C*):* Basal apodeme longer than parameres and differentially sclerotized in posterior half. Parameres broad anteriorly and tapering posteriorly to acuminate apex, with smoothly curved lateral margins and complex medial margins that are straight along most of their length but with small, curved notch at anteromedial margin and becoming bifid near posterior margin; parameres with distinctly sclerotized regions including large, comma-shaped area in posterior half. Anterior semicircular endomere extending posteriorly to anterior third of parameres with rounded apices and small medial protrusion on each side posteriorly. V-shaped small posterior endomere immediately posterior to, and between, posterior arms of anterior endomere. Small, pin-shaped medial aedeagal endomere situated at junction of basal apodeme and parameres, with larger lateral, vertically-oriented, sinuous sclerite on each side. Pseudopenis narrow and just extending to posterior apices of parameres. Subgenital plate with broadly rounded posterior and posterolateral margins, narrowing just anterior to posterior arms of basal apodeme, and widening again anteriorly; anterior margin rounded; two small lacunae near anterior margin, each with 1 long seta.

*FEMALE (*Fig. 10, Fig. 11D*) (n* = *1):* Head, thorax and abdomen as in male unless stated otherwise. Total body length of Allotype, 1.330 mm.

*Head (*Fig. 10C*):* Anterior apex slightly more curved than in male. Maximum head width of Allotype, 0.184 mm.

*Thorax (*Fig. 10C*):* Maximum width of thorax in Allotype, 0.275 mm. DPTS length of Allotype, 0.124 mm. Mesothoracic spiracle diameter, 0.025 mm.

*Abdomen (*Fig. 10C*):* Eight rows of long DCAS; row 1 with 2 setae, row 2 with 3 setae, rows 3–8 each with 4 setae; posterior curved plate with 4 shorter setae. One long DLAS on each side on segments 2, 3, 4 and 8. Six rows of long VCAS each with 4 setae. One long VLAS on each of segments 3–8. Anterior spiracle with small subtriangular paratergal plate with 1 long and 1 short PrS. Two long lateral setae on each side on segment 8.

*Genitalia (*Fig. 10, Fig. 11D*):* Subgenital plate with broadly rounded border in anterior two-thirds, then tapering abruptly to rounded posterior apex; margins uneven, especially posteriorly. Large horizontal median lacuna on subgenital plate with rounded lateral borders and bearing 4 small setae on each side. Extensive vulvar fimbriae. Gonopods indistinct; gonopods VIII with 1 small and 2 tiny setae. Patch of ∼12 curved apicolateral setae on each side, with most anterior and most posterior setae in patch distinctly thicker than others in patch. Two-three small setae on each side at abdominal apex.

#### 3.2.6 Taxonomic summary

***Type host:****Microcebus griseorufus* (Kollman, 1910) (Reddish-Grey Mouse Lemur).

***Type locality:*** Madagascar (southwest), Atsimo-Andrefana Region, Betioky District, south of Tulear at the western edge of the northern part of Tsimanampetsotsa National Park in spiny forest habitat (−24.022 S, 43.736 E, collectors: Atrefony Florent, Odilon Nicolas Germany and Julian Ehlers, collection date: October 8, 2016).

**Site of infestation:** External body surface and fur.

***Type specimens:*** 1 male Holotype (USNMENT00981954), 1 female Allotype (USNMENT00981955); U.S. National Museum of Natural History, Smithsonian Institution, Washington DC, USA.

***Etymology:*** This new species is named for Tsimanampetsotsa National Park where the Types were collected. ***Zoobank registration:*** urn:lsid:zoobank.org.act: FA4F1061–19C1-4395-8D51-388A5823296C.

### 3.3 Remarks

**Fig. 12:**
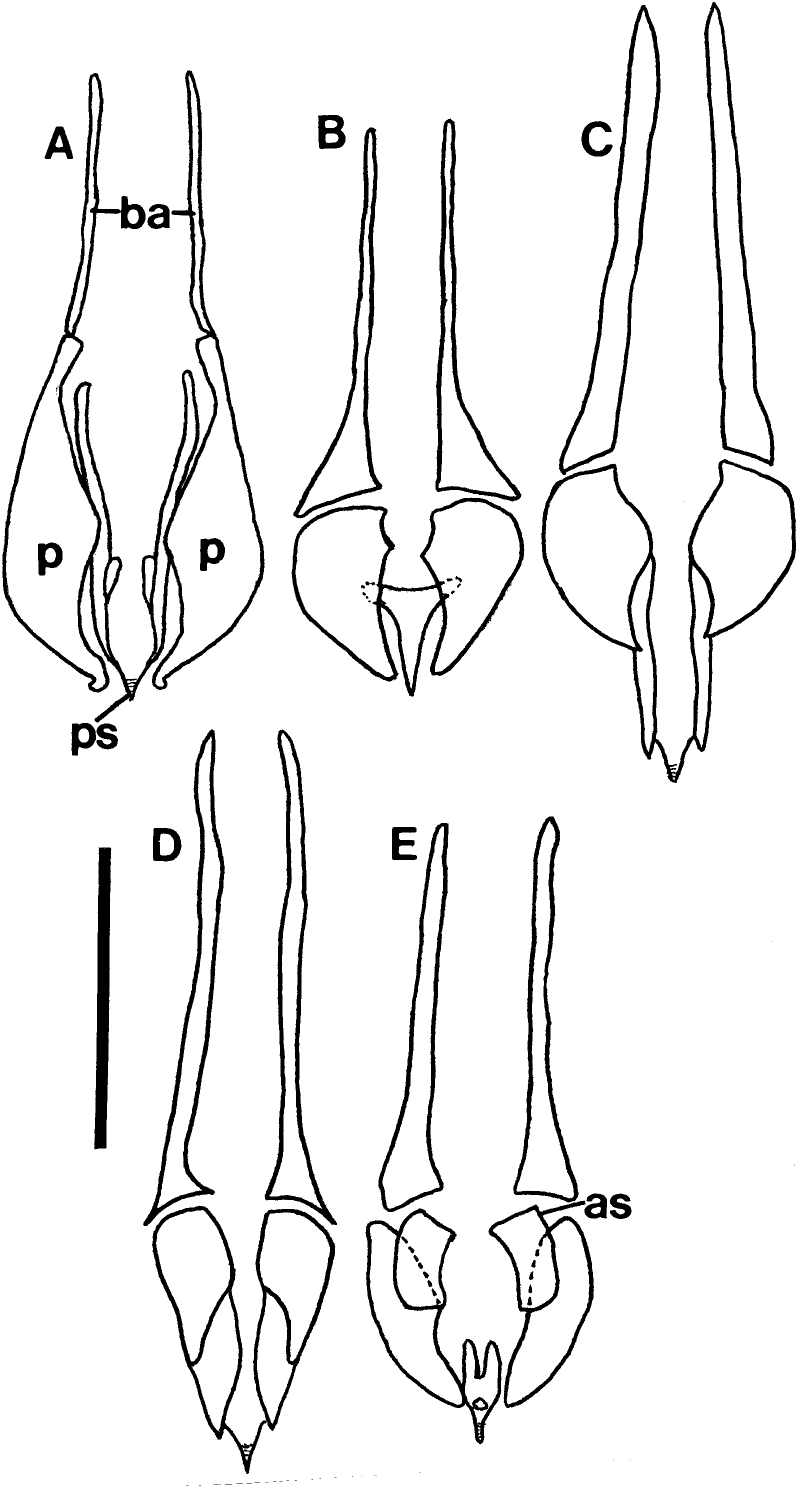
Identifying morphological characters of male genitalia for previously described species of *Lemurpediculus*, A: *Lemurpediculus petterorum* Paulian, 1958. B: *Lemurpediculus verruculosus* (Ward, 1951). C: *Lemurpediculus claytoni* Durden et al., 2017. D: *Lemurpediculus robbinsi* Durden et al., 2017. E: *Lemurpediculus madagascariensis*Durden et al. (2018). Scale bar, 0.1 mm. **Abbreviations**: **as**, accessory sclerite; **ba**, basal apodeme; **p**, paramere; ndomere; **ps**, pseudopenis.

The eight described species of *Lemurpediculus*, including the three species described in this paper, can easily be distinguished morphologically by experienced entomologists. With respect to male genitalic structures, in *L. petterorum* the basal apodeme is very narrow (about one tenth of the maximum width of the parameres) and shorter than the parameres (Fig. 12A). In all other known species of *Lemurpediculus,* the basal apodeme is much wider, with the maximum basal apodeme width approximating the maximum width of the parameres. In males of *L. gerpi* sp. nov. (Fig. 9C), *L. zimmermanni* sp. nov. (Fig. 6C), and *L. tsimanampesotsae* sp. nov. (Fig. 11C), the basal apodeme is slightly longer than the parameres, whereas the basal apodeme is more than twice the length of the parameres in all four previously described species in this genus – *L. verruculosus* (Ward) (Fig. 12B), *L. claytoni* Durden, Blanco and Seabolt (Fig. 12C), *L. robbinsi* Durden, Blanco and Seabolt (Fig. 12D), and *L. madagascariensis* Durden, Kessler, Radespiel, Zimmermann, Hasiniaina and Zohdy (Fig. 12E). In male *L.* *verruculosus* (Fig. 12B) and *L. madagascariensis* (Fig. 12E), the pseudopenis barely extends beyond the posterior apices of the parameres (to a distance less than one fifth the length of the parameres) whereas, in both *L. claytoni* (Fig. 12C) and *L. robbinsi* (Fig. 12D), the pseudopenis extends well beyond the posterior apices of the parameres (to a distance about equal to the length of the parameres). Males of *L.* *madagascariensis* (Fig. 12E) have two large accessory sclerites antero-medially between the parameres whereas males of *L. verruculosus* (Fig. 12B) lack these structures. Males of *L.* *claytoni* have broad semicircular parameres (Fig. 12C) which separates them from males of *L. robbinsi* which have narrow parameres that extend posteriorly (Fig. 12D). In *L.* *gerpi* sp. nov., the posterior endomere is very large, and wider than the maximum width of each paramere (Fig. 9C); in both *L. zimmermanni* (Fig. 6C) and *L. tsimanampesotsae* (Fig. 11C), the posterior endomere is smaller and distinctly narrower than the maximum width of each paramere. In *L.* *zimmermanni,* the middle endomere is much wider than long (Fig. 6C) whereas in *L. tsimanampesotsae* it is much longer than wide (Fig. 11C). There are many additional morphological differences between the genitalia of *L. gerpi* sp. nov.*, L. zimmermanni* sp. nov. and *L. tsimanampesotsae* sp. nov. including the distinct patterns of sclerotized areas on the posterior parameres (Fig. 6, Fig. 9, Fig. 11C).

**Fig. 13:**
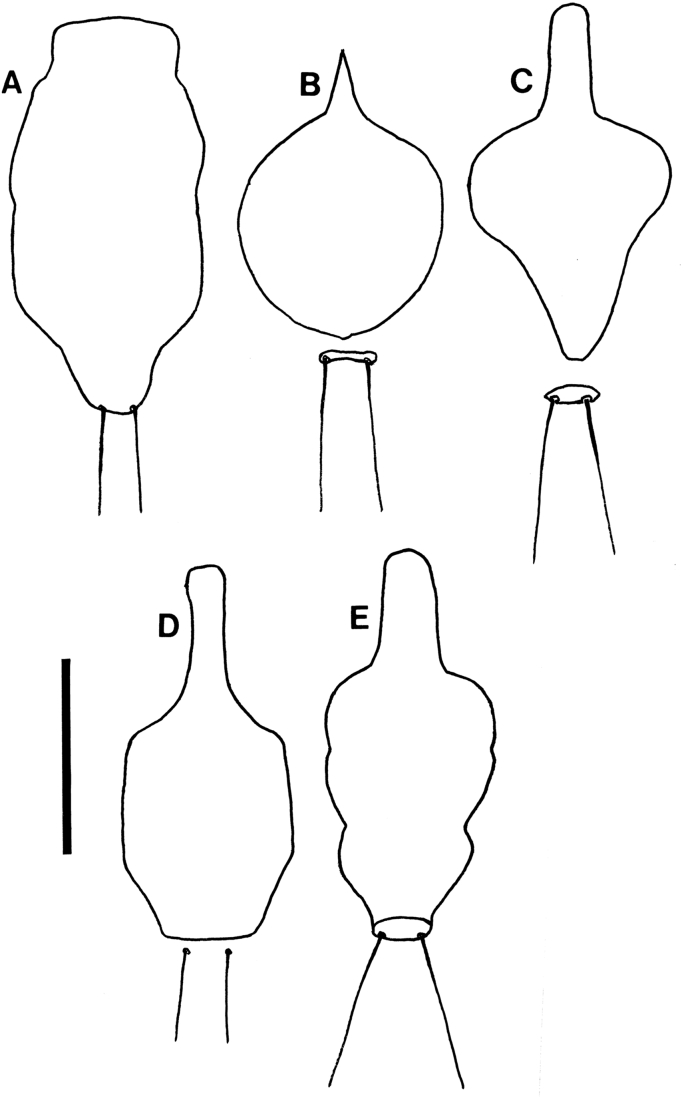
Morphology of male thoracic sternal plates and associated posterior setae for previously described species of *Lemurpediculus*, A: *Lemurpediculus petterorum* Paulian, 1958. B: *Lemurpediculus madagascariensis*Durden et al. (2018). C: *Lemurpediculus claytoni* Durden et al., 2017. D: *Lemurpediculus verruculosus* (Ward, 1951). E: *Lemurpediculus robbinsi* Durden et al., 2017. Scale bar, 0.1 mm.

**Fig. 14:**
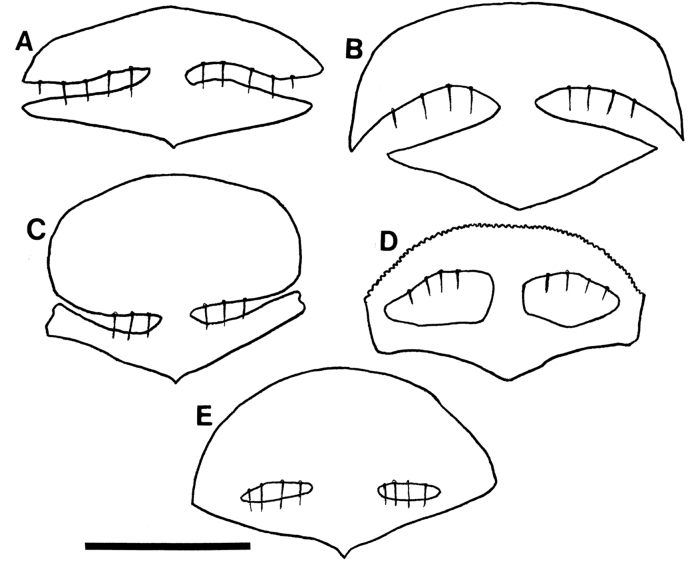
Morphology of female subgenital plates and associated setae for previously described species of *Lemurpediculus*, A: *Lemurpediculus claytoni* Durden et al., 2017. B: *Lemurpediculus robbinsi* Durden et al., 2017. C: *Lemurpediculus verruculosus* (Ward, 1951). D: *Lemurpediculus petterorum* Paulian, 1958. E: *Lemurpediculus madagascariensis*Durden et al. (2018).

*Lemurpediculus* spp. males can also be separated morphologically without examining the genitalia. Males (and females) of *L. petterorum* are unique within the genus in not having an anterior process on the thoracic sternal pate (Fig. 13A). Males of *L.* *madagascariensis* have a short acuminate (pointed) anterior process on the thoracic sternal plate (Fig. 13B); males of the other six species all have a long, terminally rounded or truncate (not acuminate) anterior process. Males of *L.* *claytoni* have an acuminate posterior process on the thoracic sternal plate (Fig. 13C) whereas males of the other five species have a rounded or truncate posterior process. In males of *L. verruculosus* (Fig. 13D)*,* the paired setae at the posterior margin of the thoracic sternal plate are much shorter than the length of the anterior process of this plate whereas in the other four species, these setae are much longer than the anterior process. In males of *L. robbinsi* the main body of the thoracic sternal plate is distinctly narrower posteriorly (Fig. 13E) whereas in the other three species it is broader posteriorly. Males of the other three species, all described in this paper, have large, paired ventral spikes on the head. In *L.* *gerpi* sp. nov., these spikes are extremely long and about half the length of the head (Fig. 9A), whereas in the other two species the spikes are much shorter (about one tenth the length of the head). In *L.* *zimmermanni* sp. nov., the posterolateral margins of these spikes are convex (Fig. 6A) whereas in *L. tsimanampesotsae* sp. nov., they are slightly concave or straight (Fig. 11A). Further, the lateral margins of the head are smoothly curved immediately posterior to the antennae in *L. zimmermanni* sp. nov (Fig. 6A), whereas in *L. tsimanampesotsae* sp. nov (Fig. 11A), there is a distinct protrusion on each side in this area (like ocular points of some haematopinid lice). Described females of all known species of *Lemurpediculus* are most easily separated morphologically based on the shape and setation of the subgenital plate. In *L.* *claytoni* (Fig. 14A), *L. robbinsi* (Fig. 14B) and *L. verruculosus* (Fig. 14C), the female subgenital plate has posterior arms and lacks lacunae whereas in the other five species arms are absent and one or two lacunae are present. The plate of *L. verruculosus* (Fig. 14C) is anchor-shaped and has three small setae on each side of the posterior border of the anterior portion. Females of both *L. claytoni* (Fig. 14A) and *L. robbinsi* (Fig. 14B) have five and four small setae, respectively, on each side of the posterior border of the anterior portion of the subgenital plate. The anterior portion of the plate is much wider than the posterior portion in *L. robbinsi* (Fig. 14B) whereas both portions of the plate are equal in width in *L. claytoni* (Fig. 14A)*.* Of the other five species, the female subgenital plate has a single horizontally elongate lacuna in *L. tsimanampesotsae* sp. nov (Fig. 11D), whereas two lacunae are present on this plate in the other four species. In *L.* *petterorum* (Fig. 14D), the paired lacunae are very large, together comprising almost half of the area of the plate; in the other three species, the paired lacunae are much smaller, together at most, comprising a tenth of the area of the subgenital plate. In *L.* *madagascariensis* (Fig. 14E), there are four small setae just anterior to each lacuna whereas in *L. zimmermanni*, sp. nov. (Fig. 6D), there are three small setae on each side.

## 4 Discussion

The present study supports a high degree of host specificity among the sucking lice of Cheirogaleidae. Generated COI and ITS1 sequences of the sampled lice generally clustered by host species, even those sampled from sympatric hosts. Only a single louse specimen from *Microcebus ravelobensis* clustered with those removed from sympatric *M. murinus*; however, it cannot be excluded that the vial was mislabelled or the host species was misidentified in the field. Nevertheless, incidental louse transfer due to sympatry is also a possibility and merits further investigation.

The observed COI and ITS1 tree topologies strongly suggest that the lice from different lemur hosts do not intermix and that those collected from *Microcebus ravelobensis, M. gerpi*, and *M. griseorufus* hosts belong to undescribed species. Their distinct morphological differences confirm that this is the case. Formal descriptions of *L. zimmermanni* sp. nov., *L. gerpi* sp. nov. And *L. tsimanampesotsae* sp. nov. are therefore included in this paper. With the description of these three new species, the total number of described sucking lice species recognized in this genus now stands at eight. Prior to 2017, only two species of *Lemurpediculus* had been described. One of these species, *L. petterorum* Paulian was stated to have been collected from “probably” *Lepilemur mustelinus* I. Geoffroy (weasel lemur, Family Lepilemuridae) (Paulian, 1958). The other seven species, including the three species described in this paper, all parasitize cheirogaleids (mouse and dwarf lemurs). Future collections of lice from other species of mouse and dwarf lemurs are expected to reveal additional species of *Lemurpediculus*. The genetic data of this study suggest the existence of two additional undescribed species parasitizing *M. danfossi* and *M. rufus*, respectively. However, only nymphal specimens were available for those lice. Formal descriptions should await the collection of adult specimens and morphological comparisons with described species. This is most easily done by comparing the shape and setation of the subgenital plate in female lice belonging to this genus and by comparing the morphology of the genitalia in males. The female subgenital plate is located on the ventral surface of the posterior abdomen and its shape can easily be determined in uncleared or cleared louse specimens using a low or high power microscope. However, the male genitalia (other than the male subgenital plate) are internal in the posterior abdomen and their morphology can only be determined in specimens that have been subjected to a clearing agent (such as potassium hydroxide) or to DNA extraction. Nevertheless, the shape of the various parts comprising the male genitalia is typically considered to be the most important criterion for morphologically distinguishing different species of sucking lice. In this respect, male *Lemurpediculus* lice have some of the most morphologically complex genitalia of any sucking lice with different structures articulating in different planes and with variously-shaped embedded sclerites called endomeres. The many different parts and shapes comprising the male genitalia in *Lemurpediculus* spp. lice facilitate species identifications.

Nevertheless, identification based on morphology alone may be challenging for non-experts, thus, a combined morphologic and molecular approach should be considered, especially as further, not yet described species may be encountered. Various molecular markers have been employed in the past to study the phylogenetics of lice (Light and Reed, 2009), but most studies employed COI and EF1α, often combining them in a concatenated dataset (Johnson et al., 2001; Smith et al., 2008; Bush et al., 2015). In the present study, EF1α alone did not offer any phylogenetic resolution for lice parasitizing different *Microcebus* spp., as their sequences were 98.0% identical. This is in contrast to previous studies, which showed that this molecular marker was suitable to distinguish between major groups of lice (Cruickshank et al., 2001) including between different species of *Pediculus* and *Phthirus* from anthropoid primates (Light and Reed, 2009). In previous studies, interspecific genetic divergence of EF1α sequences reached values of approximately 10% (Johnson et al., 2002, 2003; Light and Reed, 2009). As a protein-coding nuclear gene, EF1α evolves rather slowly compared to mitochondrial genes (Johnson et al., 2003). Thus, *Lemurpediculus* spp. of mouse lemurs may have diverged relatively recently in comparison to other lice, in line with the relatively recent divergence of their host species within the last ∼10 million to 100,000 years (Yoder and Yang, 2004; Herrera and Dávalos, 2016; Poelstra et al., 2020).

COI sequences yielded a maximum likelihood tree with well-supported terminal nodes, but a low degree of support for more basal branches, indicating that this locus is useful for differentiating between different *Lemurpediculus* spp., but perhaps less suitable for accurately resolving the phylogenetic history of the genus. ITS1 sequences yielded a generally well-supported tree, as in studies on other parasitic insects, e.g. botflies (de la Fuente et al., 2021) and fleas (Marrugal et al., 2013). However, the position of *L. claytoni* based on ITS1 was not consistent with the remaining loci, as this species appeared as a sister taxon to *L. madagascariensis*, although with low bootstrap support, possibly due to the presence of ITS1 paralogues as in other insect species (e.g. Danforth and Ji 1998; Bower et al., 2009). In contrast, *L. claytoni* and *L. robbinsi* appeared monophyletic based on EF1α, in agreement with their host associations with *Cheirogaleus* species. Therefore, ITS1 also does not seem to offer a high degree of certainty with respect to deep branching patterns. Furthermore, sequencing of this molecular marker proved difficult due to a poly-T motif, so that only a subset of the sampled lice could be included in the ITS1 and concatenated trees. This may be the reason for the lack of publicly available ITS1 sequences for Phthiraptera at the time of the analysis, and the fact that ITS1 has not been employed in previous phylogenetic studies of lice.

Due to the low degree of certainty with regard to deep branching patterns, it is difficult to make any inferences in terms of parasite-host co-evolutionary history, beyond the fact that the phylogenetic relationships of *Lemurpediculus* spp., as suggested by the present analysis, do not completely mirror the phylogeny of their hosts (Heckman et al., 2007; Weisrock et al., 2010; Hotaling et al., 2016). Incongruence between host and parasite phylogeny has been repeatedly observed in parasitic lice, and may reveal interesting insights into host evolutionary and ecological history (Johnson et al., 2002; Light et al., 2010; Sweet et al., 2020). However, further sampling of louse populations from the remaining *Microcebus* spp. and generation of whole-genome data will probably be necessary to resolve the phylogenetic history of *Lemurpediculus* spp. with certainty.

Interestingly, the COI sequence generated from a louse parasitizing *Microcebus rufus* collected at Ranomafana National Park did not cluster with *L. verruculosus* previously described from this host species at the same location. As no ITS1 sequence of *L. verruculosus* was available for comparison, this observation could not be confirmed with a second, informative locus. One possible explanation may be the existence of a second, as yet undescribed, mouse lemur species in Ranomafana National Park. The host of this louse specimen was sampled on the opposite side of the Namorona river from the *Microcebus* population where *L. verruculosus* was previously described (Durden et al., 2010) and where studies on louse infestation and movement of lice between *M. rufus* hosts were conducted (Zohdy et al., 2012, Zohdy et al., 2017). It has been suggested that rivers may create species boundaries for *Microcebus.* Future work investigating lice and *Microcebus* populations on the side of the river where this louse was recovered from may reveal distinct *Microcebus* and corresponding *Lemurpediculus* species.

Within mouse lemur species, considerable population structure between different locations can be observed (e.g. Olivieri et al., 2008). Similarly, the mitochondrial COI sequences suggested genetic separation between lice sampled from the *M. gerpi* population at Mandrizavona Sahafina compared to those from Andobo/Anjahamana, although the examined male specimens did not differ in morphological characters. Interestingly, the corresponding host populations also show genomic evidence of a long history of isolation, being geographically separated by the Rianila and Vohitra rivers (van Elst et al. in preparation). This long isolation may have led to genetic drift in hosts and lice on both sides of the rivers. As only few louse specimens were available from each of these populations, further molecular and morphologic data, including examination of female *L. gerpi* from Mandrizavona Sahafina, need to be generated to determine if all of these lice belong to the same species (*L. gerpi*), or whether a second species parasitizes *M. gerpi* at Andobo/Anjahamana.

It is likely that additional undescribed species of *Lemurpediculus* parasitize other species of cheirogaleid lemurs. In fact, there may be a rich fauna of sucking lice associated with the members of this family of small lemurs. Additional studies to collect ectoparasites from mouse and dwarf lemurs, without causing harm to the hosts, e.g., during routine capture-release missions, are therefore encouraged. Because all currently known species of *Lemurpediculus* appear to be highly host specific, these lice should be considered to be co-threatened or co-endangered together with their respective host species.

## 5 Ethics statement

All procedures were in accordance with national and international animal welfare guidelines and were authorized by the Ministére de l’Environment et du Developpement Durable (N°. 80/17/MEEF/SG/DGF/DSAP/SCB.Re, N°. 151/17/MEEF/SG/DGF/DSAP/SCB.Re, N°. 34/16/MEEMF/SG/DGF/DPT/SCBT.Re, N°. 101/11/MEF/SG/DGF/DCB.SAP/SCB, N°. 102/11/MEF/SG/DGF/DCB.SA/SCB, N°. 049/19/MEEF/SG/DGF/DSAP/SCB.Re, N°. 124/18/MEEF/SG/DGF/DSAP/SCB.Re, N°. 242/19/MEDD/SG/DGEF/DGRNE, N°. 136/16//MEEF/SG/DGF/DSAP/SCB.Re).

## Funding

This study received funding by the project INFRAGECO (Inference, Fragmentation, Genomics, and Conservation) funded by the BiodivERsA initiative of the European Community (grant no. 2015-138 to UR) and the German 10.13039/501100002347Federal Ministry of Education and Research (Bundesministerium für Bildung und Forschung) (grant no. 01LC1617A to UR), from Global Wildlife Conservation (grant #5095,008-017 5 to UR, grant #5095.020.0175 to MBB), from Houston Zoo (grant no. 05/GERP/FIN/HSZ-GERPI/18 to RR), 10.13039/100003282Primate Conservation Inc. (research grant PCI# 1299 to AK), the 10.13039/501100001655German Academic Exchange Service (10.13039/501100001655DAAD, travel grant no. 57212311 to AK), and the Gesellschaft für Primatologie (GfP, Christian-Vogel Fond to AK). Funding was awarded to SEK from the 10.13039/100000001National Science Foundation (Dissertation Improvement Grant 0961,779), the 10.13039/501100001655German Academic Exchange Service (A/09/81,743), the Animal Behaviour Society, a PEO Scholar Award, the 10.13039/100001461American Philosophical Society (Lewis and Clark Fund), Sigma Xi (National Chapter, grant G2009101504), the 10.13039/100006838American Society of Primatologists, Sigma Xi (10.13039/100007482Arizona State University chapter), the Graduate and Professional Student Association of 10.13039/100007482Arizona State University, the School of Human Evolution and Social Change of 10.13039/100007482Arizona State University, and a Dissertation Writing Fellowship from the 10.13039/100006298Graduate College of 10.13039/100007482Arizona State University. This Open Access publication was funded by the 10.13039/501100001659Deutsche Forschungsgemeinschaft (DFG, German Research Foundation) - 491,094,227 ″Open Access Publication Funding” and the 10.13039/501100005629University of Veterinary Medicine Hannover, Foundation.

## Author contributions

AS: Investigation, Formal analysis, Visualization, Writing – Original Draft. LAD: Investigation, Visualization, Writing – Original Draft. FK: Investigation, Writing - Review & Editing. AK, RR, JE, MBB, SZ, SEK: Resources, Writing - Review & Editing. SEG: Visualization. CS, UR: Conceptualization, Supervision, Project administration, Writing - Review & Editing.

## Data availability

The data underlying this article are available in the article and in its online supplementary material. Sequences generated during this study have been submitted to NCBI Genbank (for accession numbers see Table S1).

## Declaration of competing interest

None.
