# Supplementary material for: Molecular phylogenetics of the sucking louse genus Lemurpediculus (Insecta: Phthiraptera), ectoparasites of lemurs, with descriptions of three new species
Source: Int J Parasitol Parasites Wildl. 2023 Feb 9;20:138–52. doi: 10.1016/j.ijppaw.2023.02.002 (PMC9945782; doi:10.1016/j.ijppaw.2023.02.002)
Supplement: Multimedia component 1 [file mmc1.docx]

**Table S1:** Details on louse specimens and GenBank accession numbers of their elongation factor 1α (EF1α), cytochrome C oxidase subunit I (COI) and internal transcribed spacer 1 (ITS1) sequences included in the phylogenetic study. Abbreviations: *C.*: *Cheirogaleus*, *M.*: *Microcebus*; n.a.: not available due to low quality or non-amplification.

| **Louse ID** | **Host species** | **Sampling location** | **Year of collection** | **EF1α** | **COI** | **ITS1** |
| --- | --- | --- | --- | --- | --- | --- |
| LMMF2 | *M. murinus* | Ankarafantsika NP | 2017 | OP133959 | OP078650 | OP115448 |
| LMMF3 | *M. murinus* | Ankarafantsika NP | 2017 | OP133961 | OP078651 | OP115449 |
| LMMF8 | *M. murinus* | Ankarafantsika NP | 2017 | OP133962 | OP078652 | OP115450 |
| LMMM1 | *M. murinus* | Mariarano | 2017 | OP133963 | OP078653 | OP115451 |
| LMMM3 | *M. murinus* | Ankarafantsika NP | 2017 | OP133964 | OP078654 | OP115452 |
| LMMM4 | *M. murinus* | Ankarafantsika NP | 2017 | OP133965 | OP078655 | OP115453 |
| LMMM8 | *M. murinus* | Ankarafantsika NP | 2017 | OP133966 | OP078656 | OP115454 |
| LMMM9 | *M. murinus* | Ankarafantsika NP | 2017 | OP133967 | OP078657 | OP115455 |
| LMMM10 | *M. murinus* | Ankarafantsika NP | 2017 | OP133960 | OP078658 | OP115456 |
| LMMN1 | *M. murinus* | Ankarafantsika NP | 2017 | OP133968 | OP078659 | n.a. |
| LMMN2 | *M. murinus* | Ankarafantsika NP | 2016 | n.a. | OP078660 | OP115457 |
| LMMN3 | *M. murinus* | Ankarafantsika NP | 2016 | OP133969 | n.a. | n.a. |
| LMMN5 | *M. murinus* | Ankarafantsika NP | 2016 | OP133970 | OP078661 | n.a. |
| LMRF1 | *M. ravelobensis* | Ankarafantsika NP | 2017 | OP133971 | OP078662 | OP115458 |
| LMRF2 | *M. ravelobensis* | Ankarafantsika NP | 2016 | OP133972 | n.a. | OP115459 |
| LMRF3 | *M. ravelobensis* | Ankarafantsika NP | 2017 | OP133973 | OP078663 | OP115476 |
| LMRF4 | *M. ravelobensis* | Ankarafantsika NP | 2017 | OP133974 | n.a. | n.a. |
| LMRF5 | *M. ravelobensis* | Ankarafantsika NP | 2017 | OP133975 | OP078664 | n.a. |
| LMRF8 | *M. ravelobensis* | Ankarafantsika NP | 2017 | n.a. | OP078665 | n.a. |
| LMRM1 | *M. ravelobensis* | Ankarafantsika NP | 2017 | OP133976 | OP078666 | OP115460 |
| LMRM2 | *M. ravelobensis* | Ankarafantsika NP | 2016 | OP133977 | OP078667 | OP115461 |
| LMRM3 | *M. ravelobensis* | Ankarafantsika NP | 2017 | OP133978 | OP078668 | OP115462 |
| LMRM4 | *M. ravelobensis* | Ankarafantsika NP | 2017 | OP133979 | OP078669 | OP115463 |
| LMRM5 | *M. ravelobensis* | Ankarafantsika NP | 2017 | OP133980 | OP078670 | OP115464 |
| LMRM7 | *M. ravelobensis* | Ankarafantsika NP | 2017 | OP133981 | OP078671 | OP115465 |
| LMRN1 | *M. ravelobensis* | Ankarafantsika NP | 2017 | OP133982 | OP078672 | n.a. |
| LMRN2 | *M. ravelobensis* | Ankarafantsika NP | 2016 | OP133983 | n.a. | n.a. |
| LMRN3 | *M. ravelobensis* | Ankarafantsika NP | 2016 | OP133984 | OP078673 | n.a. |
| LMRN17 | *M. ravelobensis* | Ankarafantsika NP | 2017 | OP133985 | OP078674 | OP115466 |
| LMRN18 | *M. ravelobensis* | Ankarafantsika NP | 2017 | OP133986 | OP078675 | OP115467 |
| F1 | *M. rufus* | Ranomafana NP | 2007 | OP133943 | OP078688 | n.a. |
| F2 | *M. rufus* | Ranomafana NP | 2007 | OP133944 | n.a. | OP115443 |
| F3 | *M. rufus* | Ranomafana NP | 2007 | OP133945 | n.a. | n.a. |
| F4 | *C. crossleyi* | Ambatovy | 2013 | OP133946 | OP078637 | OP115442 |
| F5 | *C. crossleyi* | Ambatovy | 2013 | OP133947 | OP078639 | n.a. |
| F6 | *C. crossleyi* | Ambatovy | 2013 | OP133948 | OP078640 | OP115441 |
| F7 | *C. sibreei* | Tsinjoarivo | 2011 | OP133949 | OP078641 | OP115444 |
| F8 | *C. sibreei* | Tsinjoarivo | 2011 | OP133950 | OP078642 | OP115445 |
| M1 | *M. murinus* | Ankarafantsika NP | 2010 | OP133987 | OP078676 | OP115468 |
| N1 | *M. murinus* | Ankarafantsika NP | 2010 | OP133988 | OP078677 | OP115469 |
| N2 | *M. murinus* | Ankarafantsika NP | 2010 | OP133989 | OP078678 | OP115470 |
| N3 | *M. murinus* | Ankarafantsika NP | 2010 | OP133990 | OP078679 | OP115471 |
| N4 | *M. murinus* | Ankarafantsika NP | 2010 | OP133991 | OP078680 | n.a. |
| N5 | *M. murinus* | Ankarafantsika NP | 2010 | OP133992 | OP078681 | OP115472 |
| N6 | *M. murinus* | Ankarafantsika NP | 2010 | OP133993 | OP078682 | n.a. |
| N7 | *M. murinus* | Ankarafantsika NP | 2010 | OP133994 | OP078683 | n.a. |
| N8 | *M. murinus* | Ankarafantsika NP | 2010 | n.a. | OP078684 | n.a. |
| N9 | *M. murinus* | Ankarafantsika NP | 2010 | OP133995 | OP078685 | OP115473 |
| N10 | *M. murinus* | Ankarafantsika NP | 2010 | OP133996 | OP078686 | OP115474 |
| N11 | *M. murinus* | Ankarafantsika NP | 2010 | OP133997 | OP078687 | OP115475 |
| N12 | *C. sibreei* | Tsinjoarivo | 2011 | OP133998 | OP078638 | n.a. |
| LMGF1 | *M. gerpi* | Mandriza Sahafina | 2018 | OP133951 | OP078643 | n.a. |
| LMGF2 | *M. gerpi* | Mandriza Sahafina | 2018 | OP133952 | OP078644 | n.a. |
| LMGF3 | *M. gerpi* | Andobo | 2018 | OP133953 | OP078645 | OP115446 |
| LMGM1 | *M. gerpi* | Andobo | 2018 | OP133954 | OP078646 | OP115447 |
| LMGM2 | *M. gerpi* | Andobo | 2018 | n.a. | OP078647 | n.a. |
| LMGM3 | *M. gerpi* | Anjahamana | 2018 | OP133955 | OP078648 | n.a. |
| LMGN1 | *M. gerpi* | Mandriza Sahafina | 2018 | OP133956 | n.a. | n.a. |
| LMGN2 | *M. gerpi* | Mandriza Sahafina | 2018 | OP133957 | n.a. | n.a. |
| LMGN3 | *M. gerpi* | Mandriza Sahafina | 2018 | OP133958 | OP078649 | OP115477 |
| L32 | *M. griseorufus* | Tsimanampetsotsa NP | 2016/2017 | OP133937 | OP078701 | n.a. |
| L33 | *M. griseorufus* | Tsimanampetsotsa NP | 2016/2017 | OP133938 | OP078700 | n.a. |
| L34 | *M. griseorufus* | Tsimanampetsotsa NP | 2016/2017 | OP133939 | OP078699 | n.a. |
| L35 | *M. griseorufus* | Tsimanampetsotsa NP | 2016/2017 | OP133940 | OP078698 | n.a. |
| L36 | *M. griseorufus* | Tsimanampetsotsa NP | 2016/2017 | OP133941 | OP078697 | n.a. |
| L37 | *M. griseorufus* | Tsimanampetsotsa NP | 2016/2017 | OP133942 | OP078696 | n.a. |
| L38 | *M. griseorufus* | Tsimanampetsotsa NP | 2016/2017 | OP133931 | OP078695 | n.a. |
| L39 | *M. griseorufus* | Tsimanampetsotsa NP | 2016/2017 | OP133932 | OP078694 | n.a. |
| L40 | *M. griseorufus* | Tsimanampetsotsa NP | 2016/2017 | n.a. | OP078693 | n.a. |
| L41 | *M. griseorufus* | Tsimanampetsotsa NP | 2016/2017 | OP133933 | OP078692 | n.a. |
| MD1 | *M. danfossi* | Anjajavy | 2019 | OP133934 | OP078691 | OP115479 |
| MD2 | *M. danfossi* | Anjajavy | 2019 | OP133935 | OP078690 | OP115480 |
| MD3 | *M. danfossi* | Anjajavy | 2019 | OP133936 | OP078689 | OP115478 |
